# Enterocutaneous Fistula Secondary to Stump Appendicitis

**DOI:** 10.1155/2017/6135251

**Published:** 2017-04-03

**Authors:** Nelson Agostinho, Harinder K. Bains, Franko Sardelic

**Affiliations:** Tamworth Rural Referral Hospital, General Surgery Department, Dean Street, Tamworth, NSW 2340, Australia

## Abstract

The management of appendicitis with appendicectomy is very common in surgical practice. A recognised complication of appendicitis and appendicectomy is the formation of an enterocutaneous fistula. We present the case of a seventy-five-year-old woman who presented with an appendicocutaneous fistula on the background of an open appendicectomy performed sixty years prior to presentation.

## 1. Introduction

Appendicectomy is one of the most common surgical procedures performed in general surgical practice. Appendicitis and appendicectomy can be associated with a number of complications. Complications associated with appendicitis increase significantly when the appendix is perforated or gangrenous with periappendicitis [1]. A rare complication following appendicectomy that is associated with significant morbidity is the formation of an enterocutaneous fistula [2–4]. A fistula is defined as a communication between two epithelialised surfaces.

## 2. Case Presentation

A seventy-five-year-old female was referred by her general practitioner for management of a discharging sinus at the site of an open appendicectomy scar. The discharging sinus was associated with abdominal pain in the right iliac fossa. The patient reported that the pain commenced a month prior to presentation with the evolution of an erythematous area surrounding the scar that slowly evolved to a discharging sinus. Sixty years prior to presentation the patient had an open appendicectomy for management of clinical appendicitis. Unfortunately, due to the timing of presentation no record of that presentation or procedure existed for review. The patient reported no abdominal issues in her history after appendicectomy prior to this event.

The patient's past medical history included ischaemic heart disease for which she had cardiac stents and a cerebral artery aneurysm that had been clipped approximately 21 years prior to presentation. Regular colonoscopies were unremarkable and there was no documented or pathological history to suggest any inflammatory bowel disease. Her regular medications included aspirin, atorvastatin, and metoprolol, and she was known to be allergic to penicillin and cephalosporins.

**Figure 1:**
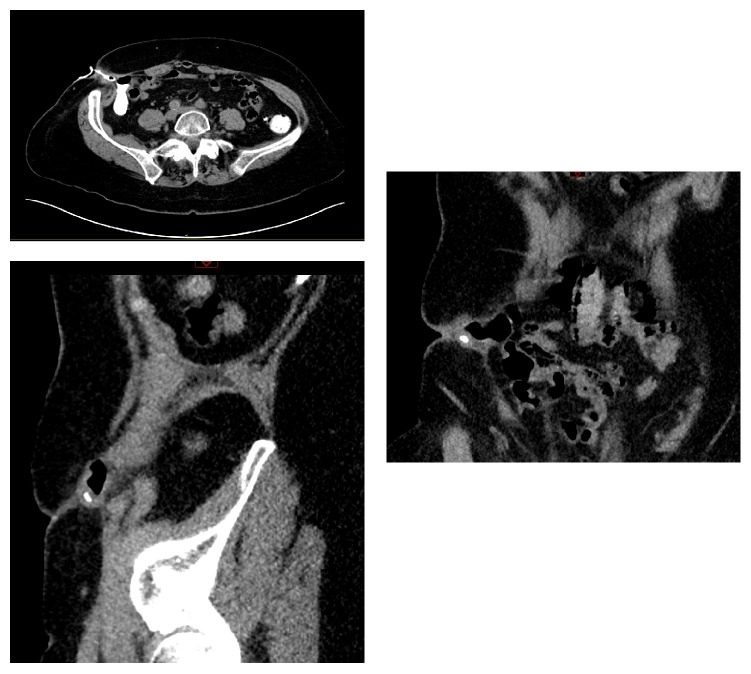
CT images of enterocutaneous fistula.

Examination of her abdomen revealed a retracted appendicectomy scar with surrounding erythema and a small sinus discharging faeco-purulent material. Her abdomen was soft and nondistended with tenderness to palpation in the right lower quadrant with associated guarding. Biochemical investigations were unremarkable. Computed tomography (CT) of her abdomen and pelvis was performed and suggested chronic tethering of the lateral wall of the ascending colon to the adjacent abdominal wall with herniation of part of the lateral wall of the ascending colon through a defect in the oblique muscles with fistulation from this hernia to the skin surface (Figure 1).

The initial treatment goal was to aim for a “controlled fistula.” A controlled fistula refers to an enterocutaneous fistula without evidence of sepsis or localised infection [5]. The patient was treated with a course of intravenous clindamycin and metronidazole for five days and was discharged home with oral equivalent two-week course. The choice of antibiotics was based on empirical therapy for common gastrointestinal flora with the patient's allergies taken into consideration. At the time of discharge there were no features of abdominal wall cellulitis, and there was no reported or elicited abdominal pain. A controlled output from the sinus remained and was managed with simple absorptive dressings.

The patient was admitted for a definitive surgical procedure several weeks after her initial presentation. The aims of the procedure included refunctionalisation of the entire bowel, resection of the fistula and any associated bowel, and secure abdominal wall closure. The procedure commenced with en bloc resection of the appendicectomy scar with careful resection around the fistula tract while tracking its course into the intraperitoneal space. The fistula was found to be intimately related to the distal caecum that was adherent to the anterior abdominal wall. The specimen was liberated from the caecum using a linear stapler and was sent for histopathological analysis. The specimen consisted of a piece of fibrofatty tissue measuring 85 × 55 × 30 mm with an overlying ellipse of skin measuring 73 × 20 mm with an attached portion of intestine measuring 12 mm in length and 35 mm in diameter. On the skin an old scar measuring 55 mm was identified, and serial sections identified a sinus filled with faecolith measuring 3 mm in diameter. Sections of the specimen were examined microscopically that showed skin with underlying adipose tissue and a portion of a stump appendix showing mucosa with underlying submucosa and muscle layer. Surrounding tissue showed fibrosis, vascular proliferation, and foreign body type giant cell reaction. There was no evidence of malignancy. Overall the features favoured a stump appendix with a fistula tract.

The patient was admitted to a surgical ward for postoperative management. This was uncomplicated and she was discharged home five days after procedure. To date the patient has had no complications.

## 3. Discussion

The formation of a postappendicectomy fistula is rare, but significant as the associated morbidities can be devastating. Major aetiological factors that contribute to postappendicectomy fistula formation include leakage from the appendiceal stump, neoplasm of the appendix and/or caecum, infective bowel conditions (e.g., tuberculosis), inflammatory bowel disease (e.g., Crohn's disease), and distal obstruction [1, 6]. The aforementioned factors are described for fistulas that form in the acute postoperative period. However, theoretically these factors may also apply to the formation of fistula at any point in the postoperative period from a stump appendix.

The relation of postappendicectomy complications to surgical technique, purse-string suture versus simple ligation of the stump, is not well established [1]. Perhaps the use of a purse-string suture may have prevented the latent formation of an enterocutaneous fistula in our patient. Histopathological analysis revealed a foreign body type giant cell reaction that may have been associated with suture material. Baldwin compared the use of purse-string suture versus simple ligation of the appendiceal stump with regard to fistula formation. He concluded that the use of a purse-string suture predisposed to fistula formation due to a number of factors that included more mobilisation of the caecum, penetration of the bowel with a needle leading to peritonitis, danger of haematoma formation from inadvertent vascular injury, necrosis of caecal wall from diminished blood supply, and an increase in postoperative adhesions [6]. Many recent studies have shown no difference between both techniques [6–9]. However, the follow-up for these studies does not extend to decades after appendicectomy.

The management of a fistula may be conservative or surgical. Nonsurgical options for a fistula include vacuum assisted closure (VAC), fistuloscopy with fibrin glue injection, and the use of monoclonal antibody pharmacotherapy in patients with Crohn's disease [1]. Surgical management of a fistula should be considered after 4–6 weeks of a sepsis-free period with adequate nutrition. Fistula tract excision and segmental resection of the involved bowel with end-to-end anastomosis are recommended [3, 10]. While nonsurgical options are considered as the first line of treatment, in our patient the surgical option was chosen for fear that the underlying aetiology was neoplastic.

Our case represents a latent, rare complication of stump appendicitis after appendicectomy. From the evidence available to us, we believe that the aetiology of our patient's fistula was secondary to stump appendicitis in a partially herniated caecum.
